# An Assessment of Incentive Versus Survey Length Trade-offs in a Web Survey of Radiologists

**DOI:** 10.2196/jmir.2322

**Published:** 2013-03-20

**Authors:** Jeanette Y Ziegenfuss, Blake D Niederhauser, David Kallmes, Timothy J Beebe

**Affiliations:** ^1^HealthPartnersBloomington, MNUnited States; ^2^Mayo ClinicDepartment of RadiologyRochester, MNUnited States; ^3^Mayo ClinicDepartment of Health Sciences ResearchDivision of Health Care Policy & ResearchRochester, MNUnited States

**Keywords:** survey methods, Internet methods, physician surveys

## Abstract

**Background:**

It is generally understood that shorter Web surveys and use of incentives result in higher response rates in Web surveys directed to health care providers. Less is known about potential respondent preference for reduced burden as compared to increased reward.

**Objective:**

To help elicit preference for minimized burden compared to reward for completion of a survey, we observed physician preferences for shorter Web surveys compared to incentives as well as incentive preference (small guaranteed incentive compared to larger lottery incentive) accompanying an electronic request to complete a survey.

**Methods:**

This was an observational study that accompanied a large Web survey study of radiology staff, fellows, and residents at select academic medical centers in the United States. With the request to complete the survey, potential respondents were offered three options: (1) a 10-minute Web survey with the chance to win an iPad, (2) a 10-minute Web survey with a guaranteed nominal incentive ($5 amazon.com gift card), or (3) a shorter (5-7 minute) Web survey with no incentive. A total of 254 individuals responded to the Web survey request.

**Results:**

Overwhelmingly, individuals chose a longer survey accompanied by an incentive compared to a shorter survey with no incentive (85% compared to 15%, *P*<.001). Of those opting for an incentive, a small, but not significant majority chose the chance to win an iPad over a guaranteed $5 gift card (56% compared to 44%).

**Conclusions:**

When given the choice, radiologists preferred a reward (either guaranteed or based on a lottery) to a less burdensome survey, indicating that researchers should focus more attention at increasing perceived benefits of completing a Web survey compared to decreasing perceived burden.

## Introduction

There is growing literature on methods to increase survey response rates in the general population [1] and also specifically among health professionals [2,3]. With respect to survey length and incentives, it is generally understood that physicians are more likely to respond to a request to complete a survey when presented with a shorter survey and when the request is accompanied with an incentive [2-5].

To our knowledge, there has been no study that has shown that longer surveys produce higher likelihood of response in a physician population. Some, however, have shown that shorter surveys perform better [2], and we know that with issue salience, length is associated with burden, which in turn is associated with increased nonresponse [3]. Specifically, in a comparison of surveys of various word lengths, Jepson and colleagues concluded that 1000 words may be a threshold of where physician likelihood of response falls off [6].

There is some evidence in physician populations that small token incentives given to all (guaranteed incentives) result in higher response rates than giving respondents the opportunity to be chosen for a larger incentive (lottery incentives). In a national survey of US nurse practitioners and physician assistants, a guaranteed $5 incentive resulted in a response rate 19.5 percentage points higher than that with a lottery incentive of the chance to win $100 [7]. Similarly a survey of emergency physicians had much greater response rates with a $2 guaranteed incentive compared to a $250 lottery incentive (56% compared to 44% respectively) [8]. An Australian study simultaneously compared the impact of survey length and lottery incentives (compared to no incentives); however, this was a general population survey. They found that a lottery resulted in higher response rates to initial mailings and that a shortened survey did not have any impact. Moreover, there was no interaction effect between a shortened survey combined with a lottery incentive [9].

However, the above research—and much of the literature on which we base decisions about how to balance minimizing perceived burden and maximizing benefit—is based on experimental designs where individuals are not given the choice between burden and reward. As such, preferences cannot be observed. Instead, individuals are randomized to one of many survey conditions with the condition producing the highest response rate being the one that is considered the most effective. Any one individual does not make a choice of what length of survey or incentive they would prefer. While the use of a randomized study design is to be lauded for its ability to isolate the impact of any given experimental manipulation, it falls short with respect to knowing what a respondent would prefer if given the *choice* of competing survey conditions thought to either minimize the burden directly (ie, shortening the length) or indirectly (ie, offering incentives). Moreover, much of what is known has been ascertained in the paper survey context; little is known about such choices when Web-based surveys are considered.

Here we present results from a study where potential respondents were able to choose among three different Web survey conditions in order to determine relative preference for burden compared to reward. Specifically, in a population of radiologists, respondents could choose to respond to a short survey with no incentive, a longer survey with a token incentive, or the same longer survey with the opportunity to win an iPad.

## Methods

This study was approved by the Institutional Review Board and determined to be exempt from informed consent requirements due to minimal risk. In late March and early April 2012, we sent an electronic request to radiology staff, residents, and fellows at 16 academic health centers in the United States asking them to participate in a survey about electronic learning resources available to them. The substantive findings of this study will be reported elsewhere (in preparation). The method of distributing the survey request differed somewhat between our home institution and other institutions. At the Mayo Clinic, the survey request was sent one time via personalized email by author BDN to all radiology residents, fellows, and staff. For the remaining institutions, our residency program director sent individualized email requests to each institution’s residency program director with a request to forward the survey links to the same population, although the forwarded surveys were not requested to be personalized. The request was sent to 209 individuals at our home institution; however, as we were unable to determine how many individuals the request was forwarded to at other institutions, we do not know how many individuals in total received the request to participate in the survey. In all, more than 1 response was obtained from 9 of the 15 institutions for which the survey requests were to be sent by program directors.

The email request described three options for completing the survey: (1) a 10-minute survey with the chance to win an iPad, (2) a 10-minute survey with a guaranteed nominal incentive ($5 amazon.com gift card), or (3) a shorter (5-7 minute) survey with no incentive. Each option was represented by a distinct link to complete the Web survey. Within each version of the survey, slight variations were present between questions for radiologists and trainees to make them applicable, such as substituting “resident” for “staff” and “your program” for “your institution”, and the third option consisted of fewer, but otherwise identical, questions.

In order to determine the extent to which different types of providers may show variable preference for length and/or incentive type, we present observed preference by key demographic variables (career stage, institutional setting, and region of the United States) and whether the individual reported using an iPad or tablet for learning or referencing radiology content. Chi-square goodness of fit tests were used to determine significance of differences. All reported differences are significant unless stated otherwise.

## Results

At our home institution (Mayo Clinic), the overall response rate was 50.2% (105 responses out of 209 requests). At the other institutions, we had an additional 149 responses for a total of 254 respondents. Because the number of survey requests sent is known only for our home institution, response rates could not be calculated overall.

A large majority (85%) of respondents preferred the combination of an incentive with a longer survey to a shorter survey without any incentive. Of those respondents choosing an incentive and longer survey, 56% chose the iPad lottery incentive and 44% the guaranteed token incentive (not significant). Overall, the iPad lottery was preferred by almost half (47%) of the respondents. See Table 1.

**Table 1:** Survey condition choice (length and incentive) overall and by practice characteristics among responding radiology residents, fellows, and staff.

Characteristics		10 minute		5-7 minute	
		$5 gift card	iPad lottery	No incentive	*P* value
Total (n=254)		38%	47%	15%	<.001
**Training**					<.001
	Resident or fellow (n=136)	43%	52%	4%	
	Staff: <3 years (n=6)	50%	50%	0%	
	Staff: 3-10 years (n=21)	19%	48%	33%	
	Staff: 10+ years (n=88)	34%	39%	27%	
	Retired (n=2)	0%	50%	50%	
**Setting**					.427
	University (n=221)	38%	46%	16%	
	Non-university (n=28)	39%	54%	7%	
	Region				.503
	Northeast (New England, etc) (n=18)	39%	44%	17%	
	Southeast (Tennessee, Carolinas, Florida, etc) (n=57)	30%	58%	12%	
	Midwest (Ohio, Illinois, Minnesota, etc) (n=173)	40%	43%	16%	
**Use of iPad/tablet for learning, referencing, or studying radiology?**					.725
	Yes (n=124)	36%	47%	17%	
	No (n=117)	41%	44%	15%	

Considering stage of training, those that were later in their career were more likely to opt for the shorter survey than were their counterparts earlier in their careers. When choosing a longer survey, these individuals more advanced in their careers were more likely to opt for the guaranteed incentive. There were no differences in preference by university as compared to nonuniversity setting or regional setting of the institution. There was also no observed difference in preference by if the individual reported present use of an iPad or tablet for studying/referencing radiology. See Table 1.

The last question on the survey itself asked respondents to disclose their address if they indeed wanted the incentive sent to them (as a matter of course with the guaranteed incentive or if they were the selected individual with the lottery incentive). Those that opted for the guaranteed incentive were somewhat more likely to not give their follow-up information. More than 8 out of 10 in the lottery condition gave their follow-up information. See Table 2.

**Table 2:** Disclosure of contact information for gift receipt by incentive condition among responding radiology residents, fellows, and staff.

	10 minute survey		
	$5 gift card	iPad lottery	*P* value
Yes, please send me a gift or enter me in the lottery	69%	84%	<.001
No thank you	22%	4%	
Missing	9%	12%	

## Discussion

Overall, the iPad incentive was the preferred choice among responding radiologists, even though it was accompanied by a Web survey that was estimated to be 3-5 minutes longer than a no-incentive condition. In fact, only 15% of respondents opted for the shorter survey option. This suggests that, among those that decide to respond to a survey, reducing burden (or perceived burden) may be less important than presenting the opportunity for individual reward via an incentive (either guaranteed or lottery-based). This pattern did not change with respect to most available practice characteristics, with the exception of career stage; those further into their career were more likely to opt for the shorter survey than their earlier counterparts. As these individuals may have more competing demands, this observation is intuitively appealing.

Observed preference for both survey length and incentive type contradict our hypotheses based on the extant literature showing that shorter surveys and guaranteed small incentives produce higher response rates. We recognize that the experimental design is different enough from past efforts so as not to cause concern about the defensibility of our a priori hypotheses or the validity of our findings. Rather, we think that there may be something about the ability to choose in and of itself that is worthy of future investigation.

In our study, we did not test the impact of choice on response rate. However, our findings lead to the question of whether some types of choice may actually benefit the survey’s outcome by empowering the potential respondent or some other yet unidentified mechanism. It is possible that offering the choice between burden and reward could alternatively decrease overall response rate as has been seen in studies of general populations that offer the choice of mode [10,11]. Either way, in this study the somewhat counterintuitive findings about preference for more survey burden in exchange for an incentive may be useful in designing future studies that could disentangle the impact of the choice in of itself from the survey condition. Ultimately, choice may be a mechanism to elicit more self-reported data from a majority of respondents.

It is also important to note that among those individuals that opted for a longer survey with an incentive, those opting for the lottery incentive were more likely to provide contact information for receipt of incentive. Fully one of five that opted for the guaranteed incentive did not choose to accept it. This could be because after completing the survey, they did not think that they needed the token reward or, perhaps, because they did not want to disclose their identity. We cannot determine the underlying mechanism from the present study design. Perhaps an investigation that is more qualitative in nature would help elucidate what the underlying mechanism(s) might be, an approach and line of inquiry recently suggested by others [3].

Our finding that among responders the iPad lottery was more preferable than the small guaranteed incentive could have important financial implications. Interestingly, if we assign the value of the iPad at $600, at the number of people who opted for this choice (120), the value was actuarially equivalent to the guaranteed incentive of $5. Of course, because the choice was offered, this could not have been predicted a priori, but it does present another potential avenue for exploration.

There are a number of important limitations to the present study that we should acknowledge to inform future work. This study was limited to radiologists at academic health centers. Due to the potentially unique composition of this population compared to other physician populations, it will be important to replicate this study design. Moreover, we do not know the denominator at institutions other than our home institution, preventing us from calculating a response rate. Incidentally, the response rate at the other institutions was likely lower than at our own (149 responses from 15 institutions) and likely due to the divergent contact protocol. However, neither the inability to calculate the response rate nor the likely lower response rate inhibits our ability to observe preference among *responders,* the primary purpose of this study. It is also important to note that all potential respondents were sent only one request to participate in the survey, whereas most physician surveys have at least two contacts. Thus it is possible that the choices we observed may be unique to early responders.

Another important limitation relates to the choices that we offered. We defined 5-7 minutes as “short” and a 10-minute survey as “long”. It is possible that the perceived burden of a survey at these two lengths was not differentiated by the providers who chose to respond. Our survey lengths were driven by the needs of the overarching study, not externally validated definitions of short and long, suggesting the need for research along these lines.

Despite these limitations, this study represents a unique contribution to our understanding of surveying this policy-relevant population. Moreover, it suggests that findings garnered from experimental designs may mask important revealed preferences by individuals when they are given the choice to weigh perceived burden and reward to survey completion.
